# Glycoprotein L sets the neutralization profile of murid herpesvirus 4

**DOI:** 10.1099/vir.0.008755-0

**Published:** 2009-05

**Authors:** Laurent Gillet, Marta Alenquer, Daniel L. Glauser, Susanna Colaco, Janet S. May, Philip G. Stevenson

**Affiliations:** Division of Virology, Department of Pathology, University of Cambridge, Cambridge, UK

## Abstract

Antibodies readily neutralize acute, epidemic viruses, but are less effective against more indolent pathogens such as herpesviruses. Murid herpesvirus 4 (MuHV-4) provides an accessible model for tracking the fate of antibody-exposed gammaherpesvirus virions. Glycoprotein L (gL) plays a central role in MuHV-4 entry: it allows gH to bind heparan sulfate and regulates fusion-associated conformation changes in gH and gB. However, gL is non-essential: heparan sulfate binding can also occur via gp70, and the gB–gH complex alone seems to be sufficient for membrane fusion. Here, we investigated how gL affects the susceptibility of MuHV-4 to neutralization. Immune sera neutralized gL^−^ virions more readily than gL^+^ virions, chiefly because heparan sulfate binding now depended on gp70 and was therefore easier to block. However, there were also post-binding effects. First, the downstream, gL-independent conformation of gH became a neutralization target; gL normally prevents this by holding gH in an antigenically distinct heterodimer until after endocytosis. Second, gL^−^ virions were more vulnerable to gB-directed neutralization. This covered multiple epitopes and thus seemed to reflect a general opening up of the gH–gB entry complex, which gL again normally restricts to late endosomes. gL therefore limits MuHV-4 neutralization by providing redundancy in cell binding and by keeping key elements of the virion fusion machinery hidden until after endocytosis.

## INTRODUCTION

Most vaccines depend on eliciting neutralizing antibodies (Zinkernagel & Hengartner, 2006). Herpesvirus carriers remain infectious despite making antibody responses. Preventing herpesvirus infections by vaccination is therefore a difficult challenge. We are using murid herpesvirus 4 (MuHV-4) to understand gammaherpesvirus neutralization. MuHV-4 binds to cells via heparan sulfate, using either gp70, a product of ORF4 (Gillet *et al.*, 2007a), or gH–gL (Gillet *et al.*, 2008a). Immune sera can block cell binding (Gill *et al.*, 2006), but they block membrane fusion poorly, allowing opsonized virions to infect macrophages and dendritic cells via IgG Fc receptors (Rosa *et al.*, 2007). Bypassing cell-binding blocks in this way is not unique to MuHV-4 (Inada *et al.*, 1985; Maidji *et al.*, 2006).

How might herpesvirus membrane fusion be blocked better? Answering this means understanding how fusion works. Virus-specific glycoproteins, such as herpes simplex virus gD, can modulate fusion (Avitabile *et al.*, 2007; Atanasiu *et al.*, 2007) and some herpesviruses can express alternative fusion complexes by using different accessory glycoproteins (Borza & Hutt-Fletcher, 2002; Wang & Shenk, 2005), but the core machinery, comprising the gH–gL heterodimer and gB (Browne *et al.*, 2001), is conserved. MuHV-4 membrane fusion is pH-dependent and occurs in late endosomes (Gillet *et al.*, 2008b). Fusion is associated with conformation changes in both gH and gB (Gillet *et al.*, 2008b, c). gB probably switches between pre- and post-fusion states, like the structurally homologous vesicular stomatitis virus glycoprotein G (Roche *et al.*, 2007), but gH is different. It switches from a gL-dependent to a gL-independent conformation in late endosomes (Gillet *et al.*, 2008c), implying that gH and gL dissociate. Yet gL^−^ virions, which constitutively express the downstream form of gH (‘gH-only’), remain infectious; indeed, they show premature rather than impaired membrane fusion (Gillet *et al.*, 2008c). It therefore appears that gH changes from gH–gL to gH-only before engaging in fusion.

Not only is gH different in gL^−^ virions: gB also shows conformational instability. This is consistent with a knock-on effect of the change in gH, as gH–gL and gB are associated in the virion membrane (Gillet & Stevenson, 2007a). A link – probably intramembrane – is maintained between gH and gB, even without gL (Gillet & Stevenson, 2007a), but any extracellular interaction must change, as the gH–gL and gH-only conformations are antigenically very different (Gill *et al.*, 2006). The gB N terminus covers part of gH–gL, and deleting it also seems to destabilize gB (Gillet & Stevenson, 2007b). This region may therefore bridge the gB and gH–gL extracellular domains.

The gB and gH conformation changes present problems for antibodies that would block membrane fusion (Gill *et al.*, 2006; Gillet *et al.*, 2006). First, antibodies must act indirectly, either by blocking conformation changes (probably the major mechanism for gH–gL) or by causing steric hindrance (probably the major mechanism for gB) (Gillet *et al.*, 2008b). Second, they must remain attached to their targets in late endosomes and compete with conformation changes that are energetically favourable at low pH. With glycan shielding (Gillet & Stevenson, 2007b) and poor immunogenicity (Gillet *et al.*, 2007b) also factored in, it is perhaps unsurprising that complete MuHV-4 neutralization is so hard.

The central roles of gL in MuHV-4 cell binding and membrane fusion suggest an additional role for it in virion neutralization. Whether gL itself is a neutralization target is unknown, but gH–gL is the major mAb-defined target on wild-type virions (Gill *et al.*, 2006). This neutralization operates downstream of cell binding, presumably by inhibiting the post-endocytic dissociation of gL from gH. Disrupting gL would remove gH–gL as a target, but could instead reveal other gH epitopes. In order to understand how gL affects neutralization, we compared the infectivity of gL^+^ and gL^−^ virions after exposure to immune sera or mAbs. Our results explain some of the resistance of wild-type MuHV-4 virions to neutralization and shed new light on herpesvirus entry.

## METHODS

### Mice, sera and mAbs.

Female C57BL/6 or BALB/c mice (Harlan UK) were infected intranasally with MuHV-4 when 6–8 weeks old, in accordance with local ethics and Home Office Project Licence 80/1992. Immune sera were collected and mAbs were derived at least 3 months later. For the latter, spleen cells were taken 3 days after an intraperitoneal virus boost and fused to NS0 myeloma cells (Köhler & Milstein, 1975). Hybrids were selected with azaserine and typed for virion glycoprotein recognition (Gillet *et al.*, 2007b). Antibodies were concentrated by ammonium sulfate precipitation, dialysed against PBS, isotyped by ELISA (Sigma) and quantified by Mancini assay (Mancini *et al.*, 1965). They are listed in Table 1.

### Cells and viruses.

BHK-21 fibroblasts (ATCC CCL-10), NMuMG epithelial cells (ATCC CRL-1636), RAW-264 macrophages (ATCC TIB-71), 293T cells (ATCC CRL-11268), CHO–gH cells (Gill *et al.*, 2006) and CHO–gp70 cells (Gillet *et al.*, 2007a) were grown in Dulbecco's modified Eagle's medium (DMEM) with 2 mM glutamine, 100 U penicillin ml^−1^, 100 μg streptomycin ml^−1^ and 10 % fetal calf serum. NS0 cells and the hybridomas derived from them were grown in RPMI medium, supplemented as for DMEM. MuHV-4 was propagated in BHK-21 cells (de Lima *et al.*, 2004). Cell debris was removed by low-speed centrifugation (1000 ***g***, 10 min) and virions were recovered from supernatants by high-speed centrifugation (38 000 ***g***, 90 min). gp70^−^ (Gillet *et al.*, 2007a), gL^−^ (Gillet *et al.*, 2007c, 2008c) and gM–enhanced green fluorescent protein (eGFP)-tagged (Gillet *et al.*, 2006) MuHV-4 mutants have been described previously. The gL^−^STOP, gL^−^DEL and gL^−^DEL-STOP mutants used here are all phenotypically equivalent. 293T cells were transfected with expression plasmids for glycosylphosphatidylinositol (GPI)-linked forms of gH, gL and gH–gL (Gill *et al.*, 2006; Gillet *et al.*, 2008a) by using Fugene-6 (Roche Diagnostics).

### Virus titres and neutralization assays.

MuHV-4 was titrated by plaque assay (de Lima *et al.*, 2004). After incubation with virus (2 h, 37 °C), BHK-21 cell monolayers were overlaid with 0.3 % carboxymethylcellulose and 4 days later fixed and stained in 4 % formaldehyde/0.1 % toluidine blue. Viruses with an eGFP expression cassette (Adler *et al.*, 2000) were alternatively titrated by eGFP expression. Cells were exposed to virus overnight in phosphonoacetic acid (100 μg ml^−1^) to prevent infection spreading. RAW-264 cells were further treated with LPS (6 h, 100 ng ml^−1^) to activate the eGFP expression cassette maximally (Rosa *et al.*, 2007). The proportion of infected cells in each culture was determined by flow cytometry. BHK-21 or NMuMG cells were infected at 0.1–0.3 p.f.u. per cell and RAW-264 cells at 1–3 p.f.u. per cell to give 20–60 % eGFP^+^ cells, eGFP titres being typically 2-fold higher than plaque titres for BHK-21 cells and 10-fold higher for BHK-21 than for RAW-264 cells. Virus titres were calculated by assuming each eGFP^+^ cell to be a single hit. For neutralization, viruses were incubated with dilutions of serum or mAb (2 h, 37 °C) before being added to the cells and assayed for infectivity as above. All sera were pooled from at least three mice. The sera within each experiment were equivalent in ELISA titre for IgG binding to Triton X-100-disrupted virions (Stevenson & Doherty, 1999).

### Immunofluorescence.

MuHV-4 virions (3 p.f.u. per cell) were exposed or not to antibody (2 h, 37 °C) then bound to cells on glass coverslips (2 h, 4 °C). The cells were then washed three times in PBS to remove unbound virions, and shifted to 37 °C to allow endocytosis. After incubation at 37 °C, the cells were fixed in 4 % paraformaldehyde (30 min), permeabilized with 0.1 % Triton X-100 (15 min) and stained with virus-specific mAbs plus Alexa 488-conjugated goat anti-mouse IgG_1_ (Invitrogen) and Alexa 568-conjugated goat anti-mouse IgG_2a_. Nuclei were counterstained with DAPI (4,6-diamidino-2-phenylindole). Fluorescence was visualized with a Leica SP2 confocal microscope and analysed with ImageJ. None of the mAbs stained uninfected cells detectably.

### Flow cytometry.

Transfected or MuHV-4-infected cells (2 p.f.u. per cell, 18 h) were trypsinized, washed in PBS and incubated (1 h, 4 °C) with MuHV-4-glycoprotein-specific mAbs, followed by fluorescein-conjugated rabbit anti-mouse IgG pAb (Dako Cytomation) or Alexa 633-conjugated goat anti-mouse IgG (Invitrogen). The cells were washed in PBS after each incubation and analysed on a FACScalibur (BD Biosciences).

### Immunoblotting.

Virions were lysed and denatured by heating (95 °C, 5 min) in Laemmli's buffer, resolved by SDS-PAGE and transferred to PVDF membranes. The membranes were probed with the ORF17 capsid antigen-specific mAb 150-7D1 plus horseradish peroxidase-conjugated rabbit anti-mouse IgG pAb (Dako Cytomation), followed by ECL substrate development (Amersham Pharmacia Biotech).

## RESULTS

### A lack of gL increases MuHV-4 susceptibility to neutralization by immune sera

**Fig. 1. f1:**
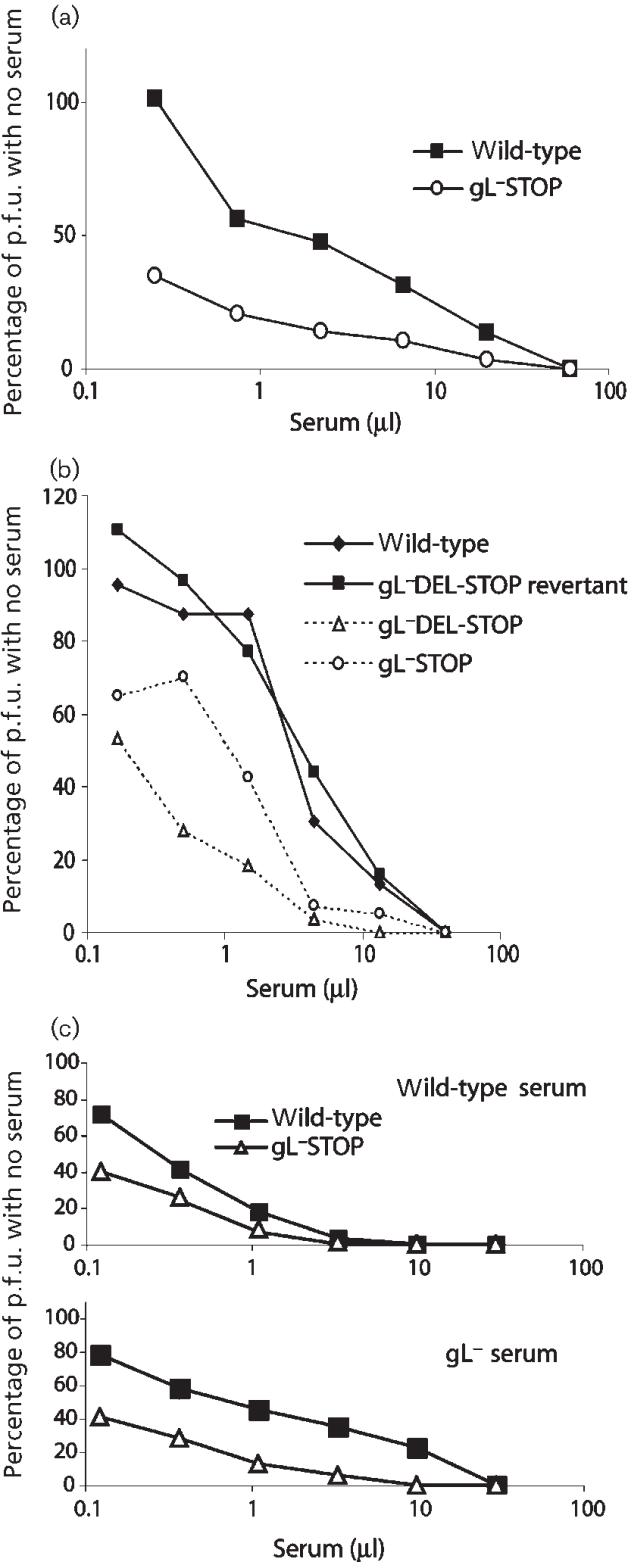
gL deficiency makes MuHV-4 vulnerable to neutralization by immune serum. (a) Wild-type or gL^−^STOP virions (100 p.f.u.) were incubated (2 h, 37 °C) with sera from BALB/c mice infected 6 months earlier with wild-type MuHV-4. The virus/serum mixtures were then plaque-assayed on BHK-21 cells. Titres are expressed relative to virus without antibody. By scoring each virus as more than or less than the mean titre of both at each serum dilution and performing a *χ*^2^ test on all data points, gL disruption was found to increase neutralization significantly (*P*<0.05). Three more serum pools all gave the same result. (b) gL^+^ (wild-type, gL^−^DEL-STOP revertant) and gL^−^ (gL^−^DEL-STOP, gL^−^STOP) virions (100 p.f.u.) were incubated (2 h, 37 °C) with immune sera from C57BL/6 mice infected 6 months earlier with wild-type MuHV-4, then plaque-assayed on BHK-21 cells. Titres are expressed relative to virus without antibody, as in (a). Both gL^−^ viruses were neutralized significantly better than either gL^+^ virus (*P*<0.05). (c) Wild-type and gL^−^ virions (100 p.f.u.) were incubated with sera from BALB/c mice infected 9 months earlier with either wild-type or gL^−^ MuHV-4, then plaque-assayed on BHK-21 cells. Titres are expressed relative to virus without antibody. The gL^−^ virus was neutralized significantly better than gL^+^ for both sera (*P*<0.05). Two more serum pools both gave the same result.

We first compared the susceptibility of gL^+^ and gL^−^ virions to neutralization by sera from mice infected with wild-type MuHV-4 (Fig. 1a[Fig f1]). gL^−^ virions were consistently more susceptible than gL^+^ virions. This applied to independently derived mutants and not to a gL^+^ revertant (Fig. 1b[Fig f1]). gL^−^ mutants were also more susceptible to neutralization by sera from mice infected with gL^−^ MuHV-4 (Fig. 1c[Fig f1]).

### The greater neutralization of gL^−^ MuHV-4 depends largely on antibodies to gp70

**Fig. 2. f2:**
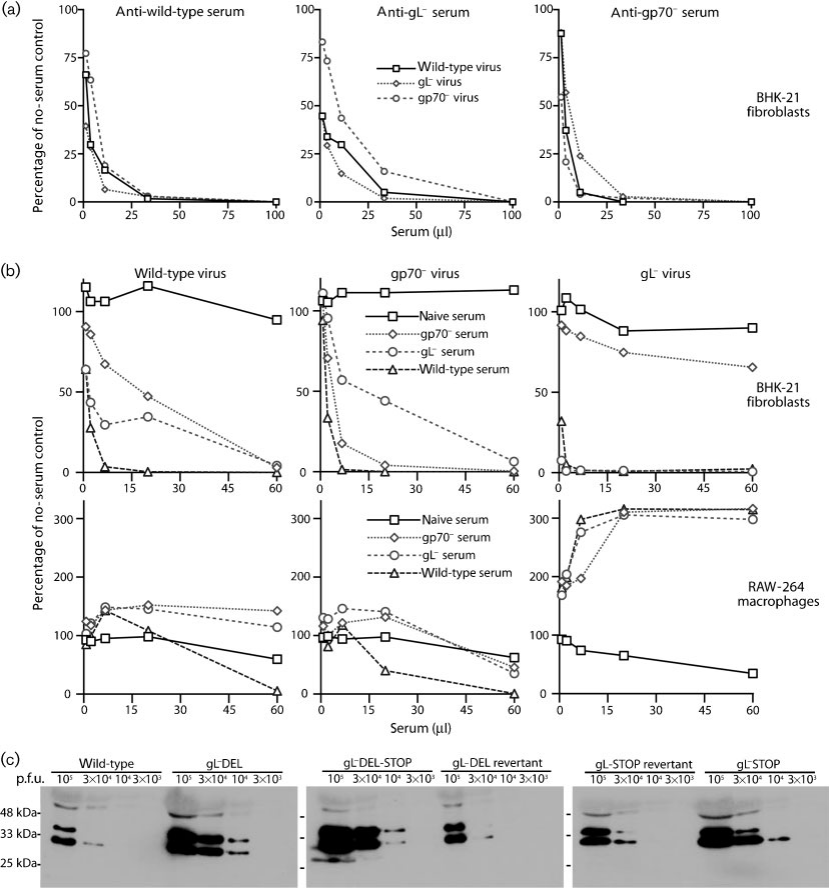
The greater neutralization of gL^−^ virions depends mainly on antibodies to gp70. (a) EGFP^+^ wild-type, gL^−^ (gL^−^DEL-STOP) and gp70^−^ virions (100 p.f.u.) were incubated (2 h, 37 °C) with sera from C57BL/6 mice infected 3 months before with wild-type, gL^−^ or gp70^−^ MuHV-4, then plaque-assayed on BHK-21 cells. Titres are expressed relative to virus without antibody. Two further experiments gave equivalent results. (b) In a similar experiment to (a), the virus/serum mixtures were split between BHK-21 cells and RAW-264 cells, and infection was measured by flow cytometry of eGFP^+^ expression 24 h later. Note that the eGFP-based assay requires more virus, so the p.f.u. (μl serum)^−1^ for each virus is 100 times higher in (b) than in (a). Each point shows the result for 10^4^ cells. Every visible difference between viruses or sera at each dilution was statistically significant by Student's *t*-test (*P*<0.001). Three further experiments gave equivalent results. The data are grouped by virus to allow comparison with naive sera. However, all the data are from one experiment and are therefore directly comparable. Thus, it can be seen that gL^−^ serum neutralized gL^−^>wild-type>gp70^−^ virus for BHK-21 cell infection. (c) gL^−^ and gL^+^ virus stocks were compared for ORF17 virus capsid content per p.f.u. by immunoblotting.

MuHV-4 binds to heparan sulfate via gH–gL or gp70. gL^−^ virions can only bind via gp70, so a possible explanation for their greater susceptibility to neutralization was that immune sera more readily blocked cell binding. We tested this by neutralizing wild-type, gL^−^ or gp70^−^ virions with sera from wild-type-immune, gL^−^-immune or gp70^−^-immune mice (Fig. 2). Sera from mice infected with knockout viruses should selectively lack antibodies against the knocked out gene product. All sera were pooled from at least three mice and had equivalent ELISA titres for detergent-disrupted wild-type virions.

We know from hybridoma analysis that even pooled mice show quite different neutralizing-antibody responses. However, the basic hierarchy between different viruses and sera was very reproducible: the neutralizations of gL^−^ virions by gp70^−^-immune sera and of gp70^−^ virions by gL^−^-immune sera were consistently poor, and eight out of eight wild-type-immune serum pools neutralized gL^−^ virions better than wild-type virions. In Fig. 2(a), whilst wild-type-immune serum neutralized gL^−^ virions only marginally better than wild-type virions, the result was clearly different from that for gp70^−^-immune serum, which neutralized gL^−^ virions less well than wild-type virions. The difference was more obvious in Fig. 2(b), where the ratios of serum to virus were lower. gp70-specific antibodies therefore appeared to be a major reason for gL^−^ virions being more susceptible to neutralization.

The converse was true, to a lesser degree, of gp70^−^ virions: they were neutralized poorly by gL^−^-immune sera, which lack antibodies to gH–gL. As MuHV-4 neutralization by immune sera correlates with a block to cell binding (Gill *et al.*, 2006) and this requires either gp70 or gH–gL, but not both (Gillet *et al.*, 2008a), it was surprising that gL^−^-immune and gp70^−^-immune sera still neutralized wild-type virions quite well (Fig. 2a, b[Fig f2]). This presumably reflects that immune sera can also neutralize in other ways. For example, some mice mount gB-specific neutralizing responses (Gillet *et al.*, 2006). Antibodies specific for abundant virion glycoproteins could also hinder infection sterically. This would explain why gL^−^-immune sera neutralized wild-type virions better than they neutralized gp70^−^ virions: gp70 is normally highly abundant and immunogenic (Gillet *et al.*, 2007b), but would be missing from gp70^−^ virions.

### Immune sera neutralize gL^−^ virions poorly for macrophage infection

Although immune sera block MuHV-4 fibroblast and epithelial-cell infections quite well, they tend to enhance dendritic-cell and macrophage infections via IgG Fc receptors (Rosa *et al.*, 2007). This reflects that Fc receptor binding allows opsonized virions to bypass blocks to conventional cell binding. The infection enhancement depends mainly on antibodies to gp150 (Gillet *et al.*, 2007b); the chief inhibitory antibodies recognize gH–gL (Gillet *et al.*, 2007d).

The greater gL^−^ virus neutralization observed for BHK-21 fibroblasts did not apply to RAW-264 macrophages (Fig. 2b[Fig f2]). Very large amounts of immune serum reduced wild-type infection, lower amounts increased it, gp70^−^ virions were similar, and gL^−^ virions showed marked infection enhancement even at serum doses virtually abolishing BHK-21 cell infection. The neutralization differences between gL^−^-immune and gp70^−^-immune sera for different virions were also greatly reduced with RAW-264 cells. These data indicated further that the strong neutralization of gL^−^ virions for BHK-21 cell infection by wild-type-immune and gL^−^-immune sera reflected mainly a better block of cell binding.

The striking enhancement of gL^−^ RAW-264 cell infection by immune sera should not be overinterpreted. gL^−^ viruses showed capsid protein : p.f.u. ratios approximately 3-fold higher than for gL^+^ (Fig. 2c[Fig f2]), i.e. gL^−^ viruses were 3-fold less infectious by plaque assay. gL^−^ and gL^+^ virus eGFP^+^ titres were proportionate to their plaque titres. Therefore, gL^−^ virions showed approximately 3-fold less infectivity for RAW-264 cells without antibody than did gL^+^ virions. The main gL-dependent infection deficits are reduced binding to BHK-21 cells (Gillet *et al.*, 2007c) and premature membrane fusion in NMuMG cells (Gillet *et al.*, 2008c). Opsonization would overcome any macrophage-binding deficit and could alleviate any premature membrane fusion by diverting virions into different endosomes. Such effects would restore gL^−^ infectivity back towards wild-type levels. Thus, it is difficult to compare degrees of gL^+^ and gL^−^ infection enhancement. Our conclusion from RAW-264 cell infections was simply that gL^+^ and gL^−^ virions showed much less difference in neutralization when their dependence on heparan sulfate for cell binding was reduced.

### gL^−^ virion neutralization maps to the gp70 N-terminal domains

**Fig. 3. f3:**
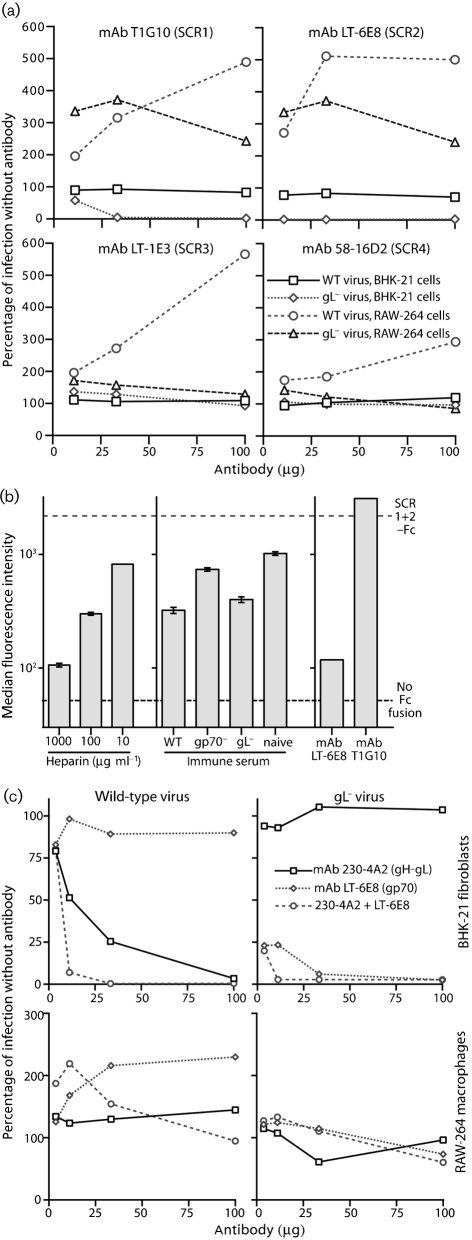
Neutralization of gL^+^ and gL^−^ virions by gp70-specific mAbs. (a) EGFP^+^ wild-type (WT) and gL^−^DEL-STOP (gL^−^) virions were incubated with mAbs specific for gp70 domains SCR1, SCR2, SCR3 or SCR4. Each virus/antibody mixture was then split between BHK-21 cells and RAW-264 cells. Infection was assayed 24 h later by flow cytometry of viral eGFP expression and is expressed relative to virus alone. Each mAb is representative of at least two recognizing the same domain. (b) An Fc fusion of gp70 domains SCR1+SCR2 was incubated with heparin, immune serum (50 μl) or mAbs (100 μg), then used to stain NMuMG cells. The dashed lines show staining with no Fc fusion or with the Fc fusion protein alone. Each bar shows the median (±standard error of the median) fluorescence intensity for 1000 cells. Each visible difference between fluorescence intensities was statistically significant by Student's *t*-test (*P*<0.001). (c) eGFP^+^ wild-type (WT) and gL^−^DEL-STOP (gL^−^) virions were incubated with mAbs specific for the heparan sulfate-binding sites of gH–gL (230-4A2) and gp70 (LT-6E8), then split between BHK-21 cells and RAW-264 cells. Infection was assayed 24 h later by flow cytometry of viral eGFP expression and is expressed relative to infection with virus alone. Each visible difference between antibodies at each dilution was statistically significant by Student's *t*-test (*P*<0.001).

Immune sera contain complex mixtures of immunoglobulin specificities and isotypes. We therefore used mAbs to define gp70-directed gL^−^ virion neutralization more precisely (Fig. 3a[Fig f3]; Table 1). gp70 comprises four short consensus repeats (SCRs 1–4) and an S/T-rich cytoplasmic domain (Kapadia *et al.*, 1999). As with the homologous protein of the Kaposi's sarcoma-associated herpesvirus (Mark *et al.*, 2006), heparan sulfate binding maps to gp70 SCRs 1–2 (Gillet *et al.*, 2007a). We identified mAb-recognition sites on gp70 by staining cells transfected with membrane-anchored gp70 C-terminal truncation mutants, as described previously (Gillet *et al.*, 2007a). The most effective gL^−^-virus-neutralizing mAb, LT-6E8, recognized SCR2; T2B11 and T1G10, which also neutralized, recognized SCR1; mAbs specific for SCRs 3–4 did not neutralize (Fig. 3a[Fig f3]). gp70-directed neutralization therefore mapped to the same domains as heparan sulfate binding.

mAb T2B11 blocks cell binding by IgG Fc fusions that contain the gp70 heparan sulfate-binding site (Gillet *et al.*, 2007a). LT-6E8 was also effective (Fig. 3b[Fig f3]). A fusion of SCR domains 1 and 2 was used here. LT-6E8 also blocked cell binding by SCR1–2–3–Fc and SCR1–2–3–4–Fc (data not shown). Inhibition of SCR1–2–Fc binding by soluble heparin is shown for comparison. Immune sera caused some non-specific inhibition of cell binding, but wild-type-immune and gL^−^-immune sera were clearly more effective than gp70^−^-immune or naive sera. Surprisingly, mAb T1G10 failed to block SCR1–2–Fc cell binding even though it neutralized gL^−^ virions. Its epitope may have a different relationship to the heparan sulfate-binding site between the Fc-linked and virion forms of gp70. Overall, gp70-directed neutralization appeared to target either heparan sulfate binding or a very closely linked function.

No gp70-specific IgG mAb blocked RAW-264 cell infection by gL^−^ virions (Fig. 3a[Fig f3]). This was again consistent with gp70-directed neutralization blocking cell binding, and therefore being unable to block infection when IgG Fc receptors provided an alternative binding route. In contrast, the gp70-specific IgM mAb T2B11 inhibits RAW-264 infection by wild-type MuHV-4 moderately (Rosa *et al.*, 2007), presumably because its bulk causes steric hindrance and RAW-264 cells lack high-affinity Fc μ binding. gp70-specific IgGs generally enhanced gL^+^ RAW-264 cell infection better than gL^−^ RAW-264 cell infection. Interestingly, SCR1/2-specific IgGs gave the best enhancement. Such antibodies may mimic the orientation of normal ligand binding and so optimally recruit the virion fusion machinery.

We then tested neutralization by mAb LT-6E8 in combination with mAb 230-4A2 (Gillet *et al.*, 2008a), which blocks heparan sulfate binding by gH–gL–Fc (Fig. 3c[Fig f3]). LT-6E8 only inhibited wild-type MuHV-4 infection of BHK-21 cells when combined with 230-4A2. 230-4A2 alone inhibited moderately, presumably because it also stabilizes gH–gL to inhibit membrane fusion; it was much more inhibitory when combined with LT-6E8 to block heparan sulfate binding by both gH–gL and gp70. RAW-264 cell infection resisted this inhibition. Thus, mAbs LT-6E8 and 230-4A2 recapitulated the hierarchical effects of immune sera (Fig. 2): 230-4A2 was analogous to gp70^−^-immune sera (no LT-6E8-type response), LT-6E8 to gL^−^-immune sera (no 230-4A2-type response) and both mAbs together to wild-type-immune sera. These data further supported the idea that immune sera inhibit fibroblast infection mainly by blocking heparan sulfate binding.

### gH-only is a neutralization target on gL^−^ virions

**Fig. 4. f4:**
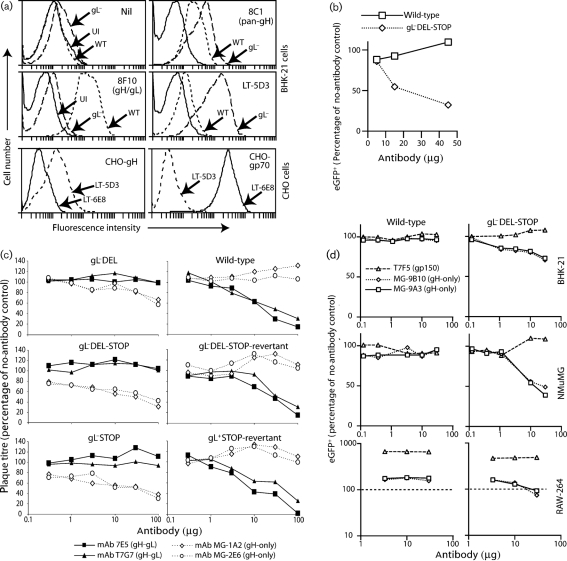
gL^−^ virus neutralization by mAbs specific for gH. (a) The specificity of mAb LT-5D3 was determined by flow cytometry of BHK-21 cells either uninfected (UI) or infected with wild-type (WT) (2 p.f.u. per cell, 18 h) or gL^−^DEL-STOP (gL^−^) (2 p.f.u. per cell, 48 h) viruses. We used different infection timings to equalize glycoprotein expression, as the cell-binding deficit of gL^−^ virions makes their cell-surface glycoprotein expression lower for a given level of infection. The background fluorescence (nil) was consequently somewhat higher for the gL^−^ infection. mAbs 8C1 and 8F10 provide staining controls. We also stained CHO cells expressing a GPI-linked gH extracellular domain without gL. CHO cells expressing gp70 and the gp70-specific mAb LT-6E8 provided staining controls. (b) eGFP^+^ wild-type or gL^−^DEL-STOP virions were mixed with mAb LT-5D3 (2 h, 37 °C), then added to BHK-21 cell monolayers. eGFP expression was assayed 18 h later by flow cytometry of 10^4^ cells. (c) gL^+^ or gL^−^ virions (100 p.f.u.) were incubated (2 h, 37 °C) with mAbs against gH–gL (T7G7, 7E5) or gH-only (MG-1A2, MG-2E6), then used to infect BHK-21 cells. Plaque titres are expressed relative to those with virus and no antibody. gH–gL-specific mAbs neutralized gL^+^ virions significantly, but not gL^−^, and vice versa for gH-only-specific mAbs. The data are from one of three equivalent experiments. (d) eGFP^+^ wild-type and gL^−^DEL-STOP virions were incubated with gH-only-specific mAbs (MG-9B10, MG-9A3) or a non-neutralizing gp150-specific control (T7F5), then split between BHK-21, NMuMG and RAW-264 cells. Viral eGFP expression was assayed by flow cytometry 24 h later, and is expressed relative to virus-only controls. The dashed line for RAW-264 cells shows this as 100 %.

Although the major neutralization difference between gL^+^ and gL^−^ virions mapped to heparan sulfate binding by gp70, this did not rule out other additional effects. In particular, the vulnerability of fibroblast infection to cell-binding blocks and the complexities of RAW-264 cell infection by opsonized gL^+^ and gL^−^ virions would have made post-binding inhibitions by immune sera hard to identify. We therefore explored gL-dependent neutralization further by testing mAbs from MuHV-4-infected mice for preferential neutralization of gL^−^ virions. We identified five mAbs: four were equivalent to LT-6E8, recognizing gp70, and were therefore not analysed further. However, LT-5D3 (Fig. 4a, b[Fig f4]) recognized gH-only, the gH antigenic form expressed by gL^−^ virions (Gillet *et al.*, 2007c). Other gH-only specific mAbs also neutralized gL^−^ virions (Fig. 4c[Fig f4]). They enhanced gL^−^ virus infection of RAW-264 cells at low doses and inhibited it, albeit weakly, at high doses (Fig. 4d[Fig f4]), a pattern similar to that of gH–gL-specific mAbs with wild-type virions (Gillet *et al.*, 2007d).

**Fig. 5. f5:**
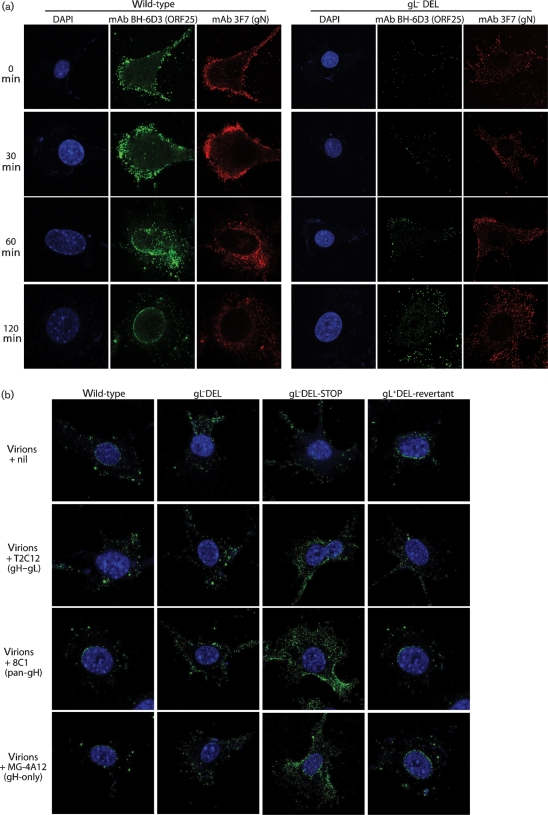
gH-directed virus neutralization visualized by immunofluorescence. (a) NMuMG cells were incubated with wild-type or gL^−^DEL virions (2 h, 4 °C), washed with PBS, incubated further at 37 °C for the times indicated, then fixed, permeabilized and stained for ORF25 (capsid) and gN. Each image is representative of >80 % of the cells examined (*n*>100). (b) The same assay was applied to gL^+^ (wild-type, gL^+^DEL-revertant) and gL^−^ (gL^−^DEL, gL^−^DEL-STOP) virions preincubated (2 h, 37 °C) with mAbs T2C12, 8C1 or MG-4A12 or with no antibody (nil). The cells were incubated with virion–antibody complexes (2 h, 4 °C), washed with PBS, incubated further to allow endocytosis (2 h, 37 °C), then fixed and stained for ORF25.

gH–gL-directed neutralization of wild-type virions occurs after binding (Gill *et al.*, 2006). This was apparent from staining cells for ORF25 major capsid antigen with mAb BH-6D3. Fig. 5(a) shows NMuMG cell entry by non-neutralized gL^+^ and gL^−^ virions, with gN co-staining for comparison. Incoming gL^+^ virion capsids migrated to the nuclear margin, whereas gL^−^ capsids remained scattered in the cytoplasm (Gillet *et al.*, 2008c). Note that BH-6D3 stains both perinuclear capsids and those still in intact virions at the cell surface. Pre-treating gL^+^ virions with mAb T2C12 (gH–gL-directed neutralization) blocked the accumulation of perinuclear capsids (Fig. 5b[Fig f5]). Virions were still endocytosed, but membrane fusion was presumably blocked (Gill *et al.*, 2006). Neither 8C1 (pan-gH, non-neutralizing) nor MG-4A12 (gH-only) inhibited gL^+^ capsid transport. gL^−^ neutralization was more difficult to analyse because gL^−^ capsids do not migrate to the nuclear margin. However, the MG-4A12 infection block clearly occurred after virion binding and endocytosis. Such a block was consistent with gH-only normally appearing only after endocytosis, with gH–Fc showing no detectable cell binding (Gillet *et al.*, 2008a) and with gH-only-specific mAbs inhibiting RAW-264 cell as well as BHK-21 cell infections (Fig. 4c[Fig f4]).

### gL^−^ virions are vulnerable to gB-directed neutralization

**Fig. 6. f6:**
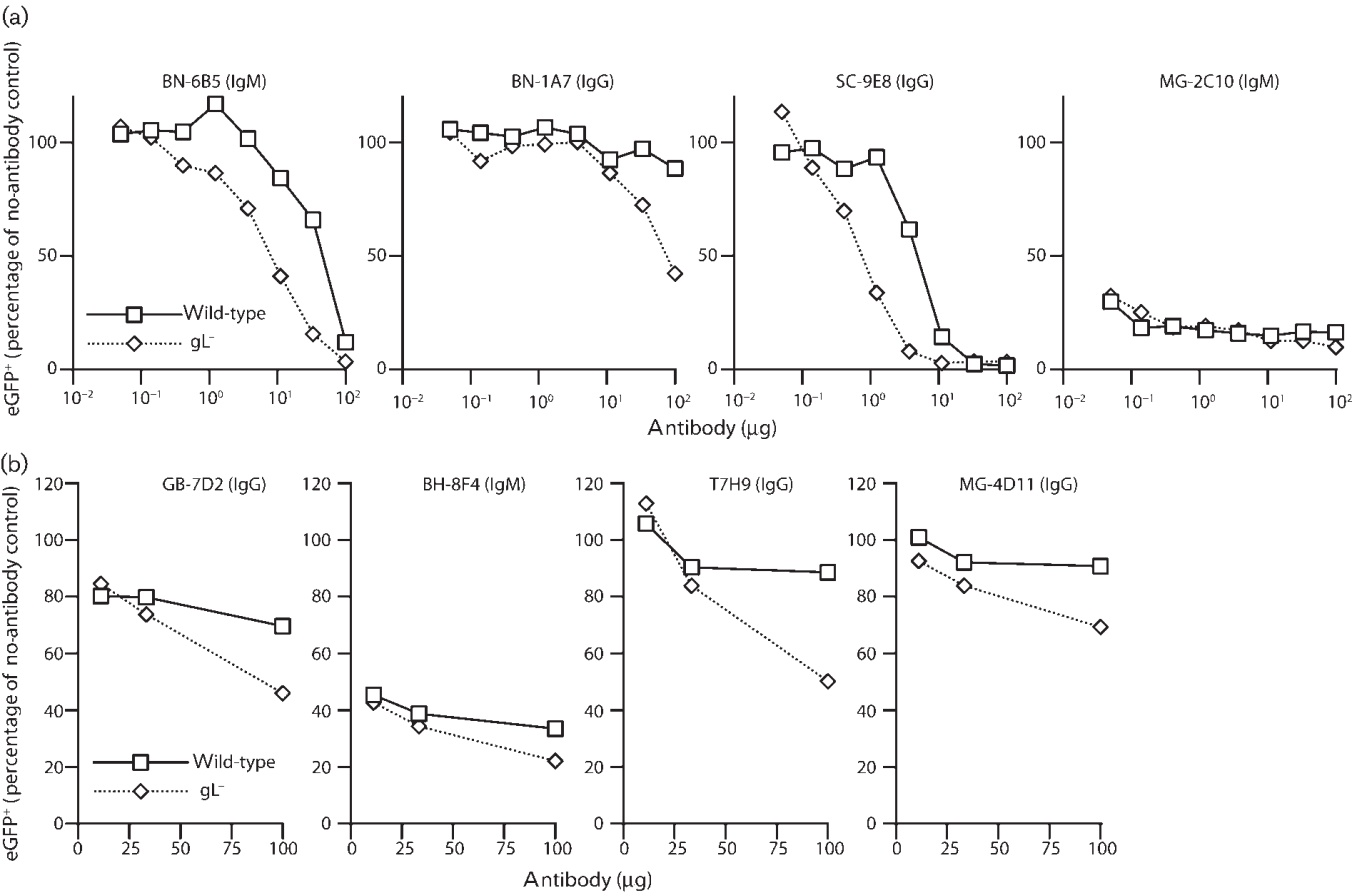
gB-directed neutralization of gL^−^ and gL^+^ MuHV-4. (a) EGFP^+^ wild-type or gL^−^DEL-STOP virions were incubated with gB-specific mAbs (2 h, 37 °C) then added to BHK-21 cell monolayers. The cells were assayed for viral eGFP expression by flow cytometry 18 h later. Each value (10^4^ cells) is expressed relative to the no-antibody control. All visible differences between viruses at each antibody dilution were statistically significant by Student's *t*-test (*P*<0.001). (b) The same assay was applied to more gB-specific mAbs.

Although none of the mAbs selected for preferential gL^−^ virion neutralization recognized gB, such a specificity could easily have been missed, as few BALB/c mice make good gB-directed neutralizing responses (Gillet *et al.*, 2006). Several established gB-specific mAbs showed gL-dependent neutralization (Fig. 6a, b[Fig f6]). Indeed, only MG-2C10, an IgM that binds to the gB N terminus (Gillet *et al.*, 2006), neutralized gL^−^ and gL^+^ virions similarly. BN-6B5, which recognizes a distinct epitope (Gillet *et al.*, 2008b), was substantially more effective against gL^−^ virions (Fig. 6a[Fig f6]). BN-1A7, an IgG_2a_ mAb whose epitope overlaps that of BN-6B5, did not neutralize wild-type virions, but had some effect against gL^−^. SC-9E8, which recognizes a different epitope in the N-terminal half of gB, was 10-fold more effective against gL^−^ virions than against gL^+^. mAb T7H9 (Fig. 6b[Fig f6]) recognizes an epitope just C-terminal to that of MG-2C10; BH-8F4 is similar to BN-6B5; GB-7D2 has not been mapped; MG-4D11 recognizes the C-terminal half of gB (Gillet *et al.*, 2006). All of these mAbs, as well as 10 others with unmapped gB epitopes, neutralized gL^−^ virions better than gL^+^. Thus, gL^−^ virions were vulnerable to gB-directed neutralization across multiple sites. This was due neither to gL^−^ virions containing less gB, nor to a conformational difference in gB (Gillet *et al.*, 2007c).

### gL itself does not appear to be a significant neutralization target

**Fig. 7. f7:**
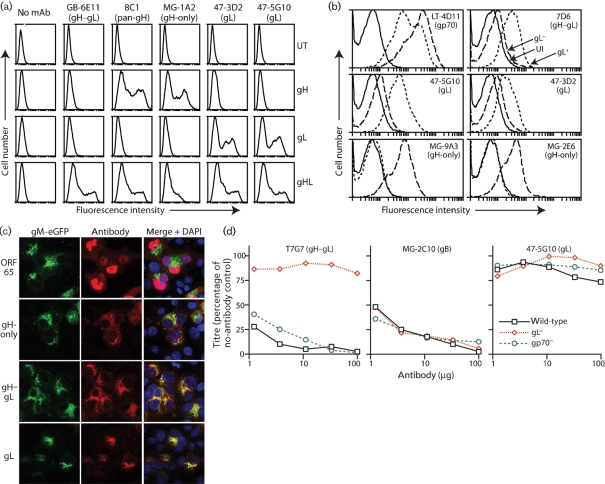
No significant neutralization by gL-specific mAbs. (a) mAbs were used to stain 293T cells either untransfected (UT) or transfected with GPI-linked forms of gH, gL or gH–gL (gHL). (b) BHK-21 cells were left uninfected (UI) or infected with wild-type (gL^+^) MuHV-4 for 18 h or gL^−^DEL-STOP (gL^−^) MuHV-4 for 48 h. As in Fig. 4(a), different timings were used to equalize the glycoprotein expression of the gL^−^ and gL^+^ infections, giving slightly higher autofluorescence for gL^−^. (c) NMuMG cells were infected with gM–eGFP^+^ MuHV-4 (2 p.f.u. per cell, 18 h), then fixed, permeabilized and stained (red) for capsid (ORF65, mAb MG-12B8), gH-only (mAb MG-9B10), gH–gL (mAb T2C12) or gL (mAb 47-5G10). gM–eGFP appears green. Nuclei were counterstained with DAPI (blue). Red–green colocalization appears yellow. (d) Wild-type, gL^−^DEL-STOP (gL^−^) and gp70^−^ viruses (100 p.f.u.) were incubated with mAbs, then plaque-assayed on BHK-21 cells. Titres are expressed relative to no-antibody controls.

The MuHV-4 gL is small – approximately 19 kDa – and probably lies close to the virion membrane (Gill *et al.*, 2006). It is therefore unlikely to be easily accessible. We derived two gL-specific mAbs. Both stained 293T cells transfected with a membrane-anchored form of gL (Fig. 7a[Fig f7]) and stained BHK-21 cells infected with wild-type but not gL^−^ MuHV-4 (Fig. 7b[Fig f7]). Immunofluorescence of infected NMuMG cells showed gL expression in a distribution consistent with the endoplasmic reticulum (Fig. 7c[Fig f7]). Our gL-specific mAbs neutralized neither wild-type nor gp70^−^ virions. Fig. 7(d) shows representative data. Also, they did not block cell binding by gH–gL–Fc (data not shown). These results were consistent with gL contributing to cell binding and membrane fusion only via gH.

## DISCUSSION

gL is a small but important component of the herpesvirus entry machinery (Roop *et al.*, 1993). The MuHV-4 gL folds gH for heparan sulfate binding (Gill *et al.*, 2006), then dissociates from gH after endocytosis to allow membrane fusion (Gillet *et al.*, 2008c). Both cell binding and membrane fusion are potential neutralization targets, and gL substantially influenced the fate of antibody-exposed virions. The major quantitative effect was on cell binding: blocking the heparan sulfate interaction of gp70 blocked the binding of gL^−^ but not gL^+^ virions. Therefore, a possible explanation for the redundancy of MuHV-4 heparan sulfate binding – something common to many herpesviruses – is antibody evasion.

Disrupting gL also made the gH-only conformation of gH a neutralization target. This supported the idea that gH-only, although downstream of gH–gL, is still pre-fusion (Gillet *et al.*, 2008c). Thus, gL limits gH-directed neutralization to inhibiting the gH–gL to gH-only transition. Antibody binding to gL-dependent gH epitopes or compound gL–gH epitopes would stabilize gH–gL. Antibodies specific for gL alone are unlikely to neutralize unless gL also changes its conformation significantly when it dissociates from gH. The recognition of both virus-infected cells and recombinant gL by our gL-specific mAbs implied that it does not. Accordingly, they did not neutralize.

Finally, gL disruption made multiple gB epitopes better neutralization targets. This presumably reflected the same molecular events as the previously observed gL-dependent conformational instability of gB (Gillet *et al.*, 2008c). However, the gB on extracellular gL^−^ virions is conformationally normal – its instability manifests only after endocytosis (Gillet *et al.*, 2008c), and gL^−^ virions were susceptible not to new gB-specific antibodies, but to those also recognizing gL^+^ virions. Therefore the greater vulnerability of gL^−^ virions to gB-directed neutralization appeared to reflect a greater exposure of its normal, pre-fusion form.

Several lines of evidence indicate that disrupting gL destabilizes gB by abolishing an extracellular interaction between gB and gH–gL. First, the gH–gL and gB extracellular domains are both unstable when expressed alone, gH–gL becoming gH-only (Gillet *et al.*, 2008a) and gB adopting mainly its post-fusion form (Gillet *et al.*, 2008b). Second, gH–gL associates with gB and, although their strongest link is probably intra-membrane (Gillet & Stevenson, 2007a), it is hard to envisage how their extracellular domains could avoid being associated too. Third, the gB N terminus hides and so presumably contacts part of gH–gL (Gillet & Stevenson, 2007b). As gB is trimeric (Heldwein *et al.*, 2006), each gB spike could contact three copies of gH–gL; if gH–gL were also multimeric, a two-dimensional lattice could form, and such clustering would allow gH–gL to hide an appreciable portion of gB. gL dissociation would then prime virions for fusion by both changing gH and revealing gB. Normally this would happen in late endosomes. A lack of gL would trigger it sooner, making pre-fusion gB accessible to antibody. Such a model would explain why most gB-specific mAbs that neutralize wild-type virions are IgMs (Gillet *et al.*, 2008b): wild-type virions express some gH-only (Gillet *et al.*, 2007c), implying that some gB is accessible; IgMs could bind one or two vulnerable gBs extracellularly, then use their remaining arms to bind newly revealed gBs in late endosomes.

A complete definition of MuHV-4 entry awaits gB–gH–gL and gB–gH crystal structures, but already some points seem clear. First, the gH–gL–gB composite presents epitopes from both gB and gH–gL. The gH–gL heparan sulfate-binding site defined by mAb 230-4A2 is not made more accessible by deleting the gB N terminus (Gillet & Stevenson, 2007b), and the N-terminal gB epitope defined by MG-2C10 was not revealed better by deleting gL. These must therefore be surface features. Second, when gL is lost, multiple epitopes on both gB and gH–gL are revealed, indicating a large-scale change. Third, the partial availability of these epitopes on wild-type virions implies that not all entry complexes are the same, perhaps because not all gH is bound to gL.

What are the implications for neutralization-based vaccines? As immunodominant viral antigens work against neutralization (Gillet *et al.*, 2007b), one priority is to identify minimal expression systems for key epitopes. An example is gH and gL fused together, which stably present native gH–gL epitopes (Gillet *et al.*, 2007d). gB-specific neutralizing IgMs are probably an unrealistic vaccine goal. However, reconstitution of the epitope of SC-9E8, a neutralizing IgG, may allow a similar approach with gB. Our increasing understanding of MuHV-4 entry makes it a suitable model to test the value of neutralization in a persistent infection.

## Figures and Tables

**Table 1. t1:** MuHV-4-specific mAbs

**mAb**	**Target**	**Isotype**	**Reference**	Neutralization*	
				**gL^−^**	**gL^+^**
T1G10	gp70 SCR1	IgG_2a_	Gill *et al.* (2006)	+++	−
T2B11	gp70 SCR1	IgM	Gillet *et al.* (2007a)	+++	−
LT-6E8	gp70 SCR2	IgG_2b_	Gillet *et al.* (2008a)	+++	−
LT-1E3	gp70 SCR3	IgG_1_	This paper	−	−
T3B8	gp70 SCR4	IgG_1_	Gillet *et al.* (2007a)	−	−
58-16D2	gp70 SCR4	IgG_2a_	Gillet *et al.* (2007a)	−	−
LT-4D11	gp70 SCR4	IgG_2a_	This paper	−	−
230-4A2	gH–gL	IgG_2a_	Gillet *et al.* (2008a)	−	+
8F10	gH–gL	IgG_2a_	Gillet *et al.* (2008a)	−	+
7E5	gH–gL	IgG_2a_	Gill *et al.* (2006)	−	++
7D6	gH–gL	IgG_2a_	Gill *et al.* (2006)	−	++
T7G7	gH–gL	IgG_2a_	Gill *et al.* (2006)	−	++
T2C12	gH–gL	IgG_2a_	Gill *et al.* (2006)	−	++
GB-6E11	gH–gL	IgG_2b_	This paper	−	++
MG-1A2	gH-only	IgG_1_	Gillet *et al.* (2007d)	++	−
MG-2E6	gH-only	IgG_2a_	Gillet *et al.* (2007d)	++	−
MG-9A3	gH-only	IgG_2a_	Gillet *et al.* (2007d)	++	−
MG-9B10	gH-only	IgG_2b_	Gillet *et al.* (2007d)	++	−
MG-4A12	gH-only	IgG_2a_	This paper	++	−
LT-5D3	gH-only	IgG_2a_	This paper	++	−
8C1	pan-gH	IgG_2b_	Gill *et al.* (2006)	−	−
47-3D2	gL	IgG_3_	This paper	−	−
47-5G10	gL	IgM	This paper	−	−
T7F5	gp150	IgG_2a_	Gill *et al.* (2006)	−	−
3F7	gN	IgG_2a_	May *et al.* (2005)	−	−
MG-12B8	Capsid ORF65	IgG_2a_	Gillet *et al.* (2006)	−	−
BH-6D3	Capsid ORF25	IgG_1_	Gaspar *et al.* (2008)	−	−
150-7D1	Capsid ORF17	IgG_2a_	Gillet *et al.* (2007d)	−	−
MG-2C10	gB	IgM	Gillet *et al.* (2006)	++	++
BN-6B5	gB	IgM	Gillet *et al.* (2008b)	++	+
BH-8F4	gB	IgM	Gillet *et al.* (2008b)	++	+
GB-7D2	gB	IgG_2a_	This paper	+	−
T7H9	gB	IgG_2a_	Lopes *et al.* (2004)	+	−
SC-9E8	gB	IgG_2a_	This paper	+++	++
MG-4D11	gB	IgG_2a_	Gillet *et al.* (2006)	+	−
BN-1A7	gB	IgG_2a_	Gillet *et al.* (2008b)	+	−

*Defined as a plaque-titre reduction of at least 50 %, and graded arbitrarily as weak (+), medium (++) or strong (+++).
